# Adding Zero-Valent Iron to Enhance Electricity Generation during MFC Start-Up

**DOI:** 10.3390/ijerph17030806

**Published:** 2020-01-28

**Authors:** Chao Li, Kang Zhou, Hanyue He, Jiashun Cao, Shihua Zhou

**Affiliations:** 1 Key Laboratory of Integrated Regulation and Resource Development on Shallow Lakes, Ministry of Education, Hohai University, Nanjing 210098, China; lichao_hhu@hhu.edu.cn (C.L.); zk2416962322@163.com (K.Z.); 2 College of Environment, Hohai University, Nanjing 210098, China; 3 Jiangsu Yuzhi River Basin Management Technology Research Institute, Nanjing 210000, China; hehanyue19940504@163.com; 4 Third Design and Research Institute, Shanghai Municipal Engineering Design and Research General Institute, Shanghai 200092, China; zhoushihua@smedi.com

**Keywords:** microbial fuel cells (MFCs), electricity generation, zero-valent iron, oxidation–reduction potential, microbial communities

## Abstract

The low power generation efficiency of microbial fuel cells (MFCs) is always a barrier to further development. An attempt to enhance the start-up and electricity generation of MFCs was investigated by adding different doses of zero-valent iron into anaerobic anode chambers in this study. The results showed that the voltage (289.6 mV) of A2 with 0.5 g of zero-valent iron added was higher than the reference reactor (197.1 mV) without dosing zero-valent iron (A4). The maximum power density of 27.3 mW/m^2^ was obtained in A2. CV analysis demonstrated that A2 possessed a higher oxidation–reduction potential, hence showing a stronger oxidizing property. Meanwhile, electrochemical impedance analysis (EIS) also manifested that values of RCT of carbon felts with zero-valent iron supplemented (0.01–0.03 Ω) were generally lower. What is more, SEM images further proved and illustrated that A2 had compact and dense meshes with a hierarchical structure rather than a relatively looser biofilm in the other reactors. High-throughput sequencing analysis also indicated that zero-valent iron increased the abundance of some functional microbial communities, such as *Acinetobacter, Ignavibacteriales, Shewanella*, etc.

## 1. Introduction

Energy crisis and environmental contamination have been pushing researchers to search for new kinds of sustainable, renewable, and clean energy [1]. Microbial fuel cells (MFCs) are a green bioenergy technology, which are considered as a newly bioelectrochemical device that can convert available organic matters into electricity by exoelectrogenic bacteria attached on the anode [2,3]. Therefore, MFCs offer a new opportunity for simultaneous wastewater treatment and electricity generation [4]. At present, many efforts have been made to improve the performance of MFCs [5,6,7] and many works have focused on the microbial community composition of the anode biofilm [8]. However, little attention has been devoted to the effect of metal elements on the biofilm during start-up in MFCs.

In recent years, many metals and their oxides were used to modify the cathode or anode of MFCs in an attempt to improve the ability to transfer electrons in reactors. In MFCs, the pass-way of the anode’s extracellular electrons transfer can be divided into direct electron transfer and indirect electron transfer [9,10,11]. The former is transferred by the microbial extracellular cytochrome or “nanowires” while a soluble redox mediator is needed in the latter. Furthermore, transfer mediators can be added exogenously or endogenously [12,13], and microbes use their own redox secretion or extraneous substances to facilitate electron transfer extracellularly, and then to the electrode surface [14,15]. The addition of metal elements can not only improve electrical performance directly but also play an important role in the culture of exoelectrogenic bacteria.

Studies show that the electrical performance of MFCs depends largely on the number of exoelectrogenic bacteria enriched on the anaerobic biofilm, most of which belong to iron-reducing bacteria (IRB). Moreover, a further study found that adding Fe (III) oxide to MFCs could enrich exoelectrogenic bacteria and also enhance the electrical performance of reactors. For example, Zhang et al. [16] pointed out that the addition of Fe (OH)_3_ enhanced both anaerobic digestion and anodic oxidation, resulting in an effective mineralization of volatile fatty acids (VFAs), which was beneficial for improving anodic oxidation and enhancing microorganisms’ growth. Also, Wu et al. [17] demonstrated that Fe (III) supplemented into MFCs resulted in increased electricity generation by *Shewanella oneidensis* MR-1.

Zero-valent iron, the most basic form of iron, has seldom been used directly in the research of MFCs and it is in a doped or oxidized state. However, zero-valent iron, ferrous, or ferric iron is inextricably linked by accepting and losing electrons in redox reactions. It was assumed that zero-valent iron was likely to enhance the conversion of propionate to acetate because it could serve as an electron donor in microbial metabolism [18] and promote the activities of key enzymes in the acetogenesis process [19,20]. Interestingly, it is generally believed that acetic acid [21] could act as an appropriate substrate for exoelectrogenic bacteria, thus the power generation of MFCs is greatly improved.

However, the mechanism of zero-valent iron obtained in MFCs during start-up has been rarely reported. To gain insight into understanding the effects of zero-valent iron on MFC, the electron transfer property, anode biofilm formation, and biological community were investigated and analyzed in MFCs with different amounts of zero-valent iron addition in this study.

## 2. Materials and Methods

### 2.1. Experimental Device

**Figure 1:**
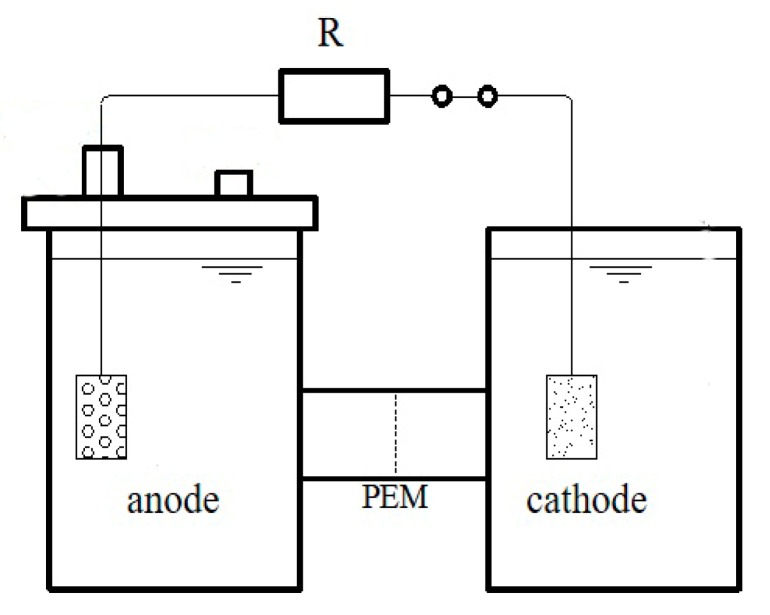
Schematic diagram of the MFC reactor used during the experiment.

H-type MFC devices consisted of two-cylinder transparent polyacrylic plastic bottles (18 cm × Φ12 cm), separated by a proton exchange membrane (PEM) (Nafion 117, Dupond, Hayward, CA, USA), whose inner diameter was 2 cm. Each chamber has a carbon felt electrode (5 cm × 6 cm × 0.2 cm, US Morgan). The electrode space is 15 cm and connected with an external resistance of 1000 Ω as shown in Figure 1. Before experiments, the MFC components were sterilized with an autoclave at 121 °C 15 min. All MFCs were operated in a temperature-controlled room at 25 °C.

### 2.2. Experimental Methods

Experiments were conducted in batch mode with four parallel two-chamber MFCs containing four different concentrations of zero-valent iron: 0.1 g of zero-valent iron added (A1), 0.5 g of zero-valent iron added (A2), 1 g of zero-valent iron added (A3), and no zero-valent iron added (A4).

The two chambers, both filled with electrode medium (pH 7.0), contained: (NH_4_)_2_SO_4_ (0.56 g/L), KCl (0.13 g/L), NaH_2_PO_4_ (4.22 g/L), Na_2_HPO_4_ (2.75 g/L), MgSO_4_·7H_2_O (0.2 g/L), and 1 mL/L of trace elements solution, containing: H_3_BO_3_ (2 mg/L), FeCl_2_·4H_2_O (2 mg/L), EDTA (2 mg/L), ZnCl_2_·4H_2_O (0.4 mg/L), MnCl_2_·4H_2_O (0.8 mg/L), CuCl_2_·2H_2_O (0.2 mg/L), (NH4)_6_MO_7_·4H_2_O (1.1 mg/L), and NiCl_2_·6H_2_O (1mg/L). Sodium acetate was controlled at COD (Chemical Oxygen Demand) of 800 mg/L in the anodic solution.

The sludge was obtained from the sedimentation tank of a municipal sewage plant in Nanjing (China). After removing large debris, the volatile suspended sludge (VSS) was 4.4 g/L and total suspended sludge (TSS) was 8.0 g/L.

The anaerobic digester sludge was inoculated in the anode compartment (15%, *v/v*) as the biological catalyst, whereas the cathode compartment was continuously aerated with air.

### 2.3. Electrochemical Analysis

The voltage was measured by a voltmeter, and polarization curves for the MFCs were generated by changing the external resistance in the range 10–10,000 Ω. Power density was calculated using the formula, *p* = UI/A, where U denotes the voltage between the anode and cathode, I denotes the current calculated from Ohm’s law, and A denotes the area of the anode carbon felt.

Cyclic voltammetric characterization of the oxide electrode was performed in the potential range of −0.7 to 0.7 V with respect to the calomel electrode (NCE), and Pt wire was used as the counter electrode in the phosphate buffer. The impedance spectra of the anode film were recorded in the frequency range from 100 kHz to 10 mHz with the alternating current (AC) amplitude of 10 mV, and the data collected were analyzed by using Zsimpwin software [22].

### 2.4. Chemical Analysis

TSS, VSS, and COD were conducted in accordance with Standard Methods for the Examination of Water and Wastewater [23]. The morphology of the four anodes was subjected to scanning electron microscopy (SEM) by cutting off 0.5 cm × 0.5 cm × 0.2 cm pieces.

### 2.5. High-Throughput Sequencing (Miseq)

High-throughput sequencing of the 16S rRNA gene (Miseq, Illu-mina) was conducted for further analysis of the key four sludge samples. The sequencing and data analysis were performed by the Zhongyijinda Analytical & Testing Co., Ltd (Jiangsu; China). The heatmaps and cluster analyses were obtained using the R programming language (R-3.1.0).

## 3. Results and Discussion

### 3.1. Effect of Zero-Valent Iron on Voltage of MFCs during Start-Up

**Figure 2:**
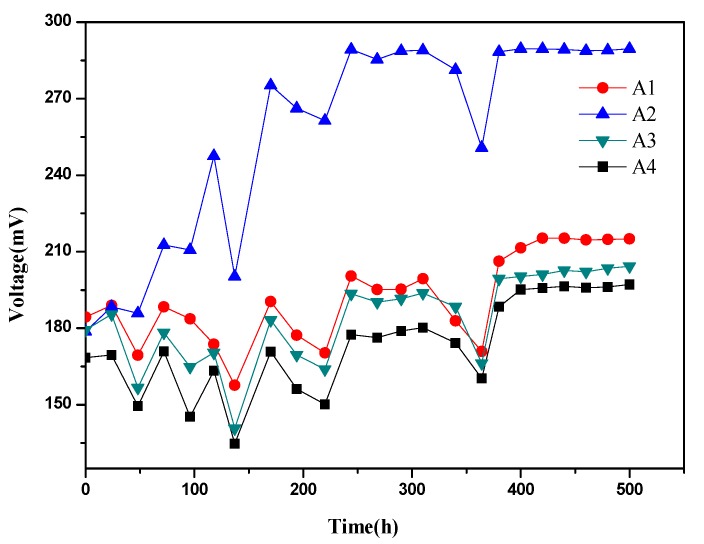
Cell voltage as a function of time with an external resistance of 1000 Ω during start-up (A1: 0.1 g zero-valent iron; A2: 0.5 g zero-valent iron; A3: 1 g zero-valent iron; A4: no zero-valent iron).

The voltage output of four groups increased slowly at first and finally became stable in MFCs supplemented with different concentrations of zero-valent iron, as shown in Figure 2. The voltage of A2 was the highest, which could reach up to 289.6 mV, and the voltages of A1 and A3 had no big difference, with a maximum of 215.3 and 204.3 mV, respectively. Surprisingly, the voltage of A4 could only reach the peak of 197.1 mV. In addition, it can be found that A2 took 400 h to reach the voltage platform while A1 took 420 h and A3 and A4 still had a rising tendency after 500 h, suggesting that the addition of zero-valent iron accelerated the start-up of the reactors. Zero-valent iron substantially affected the voltage production as well as the voltage platform during the start-up period of MFCs, and the highest voltage was observed with the appropriate concentration range.

### 3.2. Effect of Zero-Valent Iron on Power Density of MFCs during Start-Up

**Figure 3:**
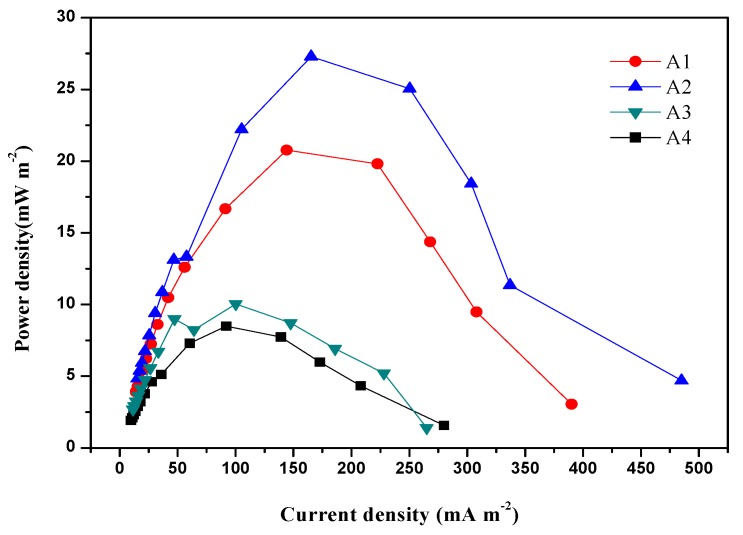
Power–density curves during start-up (A1: 0.1 g zero-valent iron; A2: 0.5 g zero-valent iron; A3: 1 g zero-valent iron; A4: no zero-valent iron).

Figure 3 illustrates the power density of the MFC with a varying zero-valent iron concentration. MFCs supplemented with zero-valent iron during start-up showed higher power densities than that obtained in the control without zero-valent iron. The maximum power density of 27.3 mW/m^2^ in the presence of 0.5 g of zero-valent iron was obtained, compared to 8.5 mW/m^2^ without zero-valent iron. Meanwhile, A1 delivered a maximum power density of 20.8 mW/m^2^ and A3 reached the peak of 10.0 mW/m^2^. Furthermore, the power density was decreased when the zero-valent iron concentration further reached 1.0 g. An increase in the power density occurred at first and then decreased as the current density increased because of the polarization of internal resistance. The results showed good consistency with the observed voltage, indicating that zero-valent iron could greatly enhance the power generation of MFCs at low concentrations; however, the reaction would be inhibited at higher concentrations. Jia et al. [24] also verified that the addition of ZVI to the digestion could retard excessive acidification by promoting butyric acid conversion and accumulating direct interspecies electron transfer simultaneously to enhance the biosensor’s performance under a moderate amount of ZVI. The reason for this phenomenon could be explained as follows. On the one hand, Fe^2+^ (Fe^0^ + 2H^+^ = Fe^2+^ + H_2_) would be released when supplemented with a moderate dose, which changes the iron’s strength inordinately and compresses the electric double layer of sludge effectively [25,26]. On the other hand, zero-valent iron would decrease reactors’ ORP and reduce the accumulation of propionate, which facilitates the conversion of macromolecular acids to a smaller molecule [27].

### 3.3. Electrochemical Activity of MFCs

**Figure 4:**
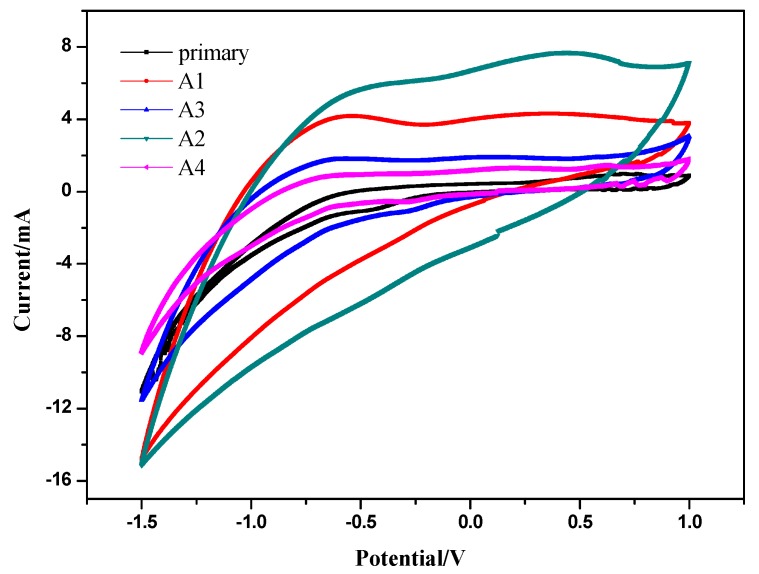
CV curves of anodic carbon felt of MFCs (A1: 0.1 g zero-valent iron; A2: 0.5 g zero-valent iron; A3: 1 g zero-valent iron; A4: no zero-valent iron).

Cyclic voltammetry (CV) enables direct detection of redox signals, senses potential differences across the interface, and helps elucidate electrochemical reactions occurring at electrode surfaces [28], so CVs were tested during stable current generation to evaluate the importance of zero-valent iron on anodic electrochemical behavior. A single peak showing no retracing trend indicated this process was irreversible, as shown in Figure 4. The rate of ipa/ipc of these two peak currents was unequal to 1, which also confirmed this judgment. Furthermore, the gap of the peak potential between the anode and cathode was aggregated, illustrating that the irreversible degree became larger, namely, ΔEp >> Upa−Upc ≈ 0.056/n [29,30].

No big difference in the irreversible degrees between each reactor supplemented with different doses of zero-valent iron was found, but there were still a couple of weakly defined redox peaks at −0.6 (A1) and 0.5 V (A2), and the CVs of the zero-valent iron added exhibited a broader current range, suggesting zero-valent iron might produce redox mediators. As we all know, the redox potential can reflect the macroscopic oxidation–reduction property of substances in aqueous solution; the higher the oxidation–reduction potential, the stronger the oxidation ability. In Figure 4, the oxidation–reduction potential of A2 was higher than A1, showing that the oxidation ability of A2 was stronger and the solution was oxidizing. In addition, the areas of five closed redox curves varied dissimilarly, specifically A2 > A1 > A3 > A4 > primary carbon felt. It has been pointed out that the area of CV can reflect the amount of electricity exchanged between the oxidation and reduction reactions of an electro-active material, which can also demonstrate the polarization state of the internal electrode as well as the utilization of active substance [31,32]. So, it can be inferred that A2 has the most electro-active substances and its utilization rate of exoelectrogens was relatively higher.

Combined with the analysis above, A2, which had a suitable dose of zero-valent iron added, exhibited excellent electrochemical activity. The positive oxidation potential illustrated the reaction was more oxidizing and the metabolism of exoelectrogens on the surface of the electrode was more active. However, the oxidation peak of A3 was suppressed, because the addition of excessive zero valent iron inhibited the partial oxidation of anode microorganisms in the MFC, which probably weakened the redox reaction. Therefore, it was possible that the overall performance of MFCs could be improved by choosing an optimal concentration of zero-valent iron in the anode solution.

EIS (electrochemical impedance spectroscopy) was used to measure anode properties, such as the internal resistance and coating layers [33]. In MFCs, the performance of the anode is mainly affected by electrode reaction kinetics and mass diffusion. So, in order to figure out the effects of different doses of zero-valent iron on the internal transfer impedance and distribution of electro-active biofilm, the EIS of different electrodes was tested. As we all know, the charge between the surface of the electrode and contacted electrolyte solution was the opposite, forming an electric double layer capacitance. Meanwhile, electron transfer resistance also formed due to asynchronization of the electronic transfer, and differences between the electronic conductivity and solution ion conductivity when electrons were transferred on the interface of electrode/electrolyte. What is more, a diffusion layer trend is formed by solution ions under the effect of the voltage, and the electrolyte solution itself also has a certain impedance.

**Figure 5:**
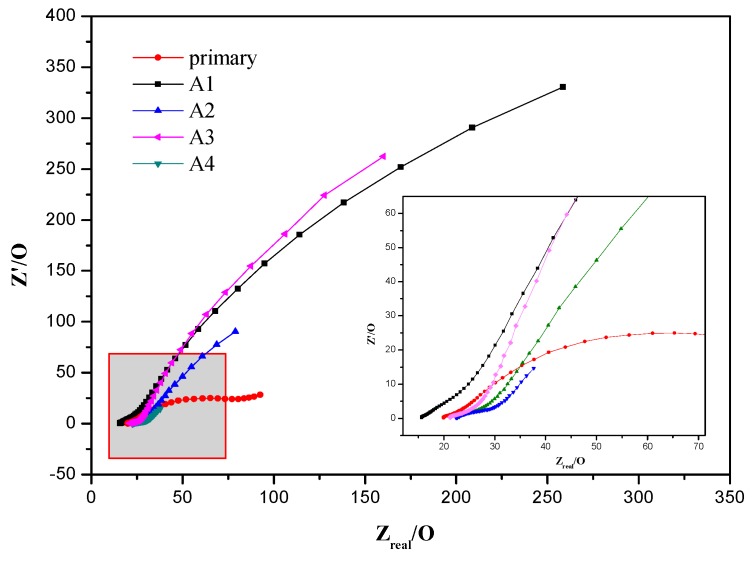
Impedance spectra (Nyquist) of anodic carbon felt of MFCs (A1: 0.1 g zero-valent iron; A2: 0.5 g zero-valent iron; A3: 1 g zero-valent iron; A4: no zero-valent iron).

Nyquist plots are shown in Figure 5. It could be seen from the curves that all five carbon felts have the shape of a circular arc, representing resistance when electrons transfer inside it, and a straight line close to 45°. According to polarization theory, diffusion impedance exhibits a straight line at low frequency, suggesting that Warburg impedance, which is caused by the diffusion of oxygen in electrolyte solution outside the electrode, is formed and has the characteristic of semi-infinite diffusion. Th arc diameter is relatively larger in an open circuit because it is related to the exchange current density of the oxygen reduction reaction [34,35].

The experimental spectra were fit into an equivalent circuit according to Wagner to estimate the impedance data quantitatively using Zsimpwin software [22]. Values of the circuit elements are given in Table 1. Especially, the equivalent circuit included a charge transfer resistance (R_CT_), a constant phase element © in parallel, and a Warburg impedance (W). The kinetics of the electrochemical reaction could be summarized from the faradic impedance, including the combination of R_CT_ and W [36].

In this electrochemical system, the arc appearing at high frequency represented R_S_ (ohmic resistance) since the structure of the working electrode carbon felt was porous. The fitted data suggested that R_S_ were close to each other [37,38], with an average value of 22.43 ± 2.18 Ω under different doses of zero-valent iron. This was because R_S_ mainly depends on the configuration of the reactor while the catalytic effect is not significant. In addition, there were two other processes in the solution: Charge transfer and mass transfer. R_CT_ (charge transfer internal resistance) is caused by the energy activation barrier, which needs to be overcome during an electro-chemical reaction, while mass transfer resistance indicates the effect of mass diffusion on the electrode and it can be defined as semi-infinite diffusion by the shape in the study.

R_CT_ in Table 1 showed that the R_CT_ of carbon felts with zero-valent iron supplemented were generally low while values with no zero-valent iron supplemented were sharply increased. After a period of domestication, a layer of biofilm attached, with zero-valent iron forming and then adsorbing on the electrode surface so that electrons produced by exoelectrogens could be transferred by this biofilm, thereby reducing R_CT_. R_CT_ in A2, which had 0.5 g of zero-valent iron added, had the lowest value (0.01 Ω) while A4 (33.84 Ω) with no zero-valent iron added had the highest (except primary carbon felt). Combined with SEM above, the biofilm of A2 was relatively enriched and electro-active microorganisms continuously adhered to the electrode, which hastened the electron transfer rate of carbon felts and reduced their resistance. Hence, it was possible to confirm that the high values of the voltage and power density precisely contributed to the low R_CT_, which improved the electron transfer efficiency and enhanced the electrochemical performance. However, W (Warburg impedance) of A2 was relatively high because the biofilm enriched in A2 was mature, abundant, and thick. Correspondingly, there were less electro-active microorganisms in A4, so the resistance was higher during the mass transfer process and mass diffusion was more difficult, which caused the high value of R_CT_. In addition, it was found that the second arc of primary carbon felt was biased and it was not a strictly semi-infinite diffusion, appearing as the shape of an arc because of the lack of biofilm enrichment and poor conductive properties.

### 3.4. SEM Images of the Anode Surface of MFCs

**Figure 6:**
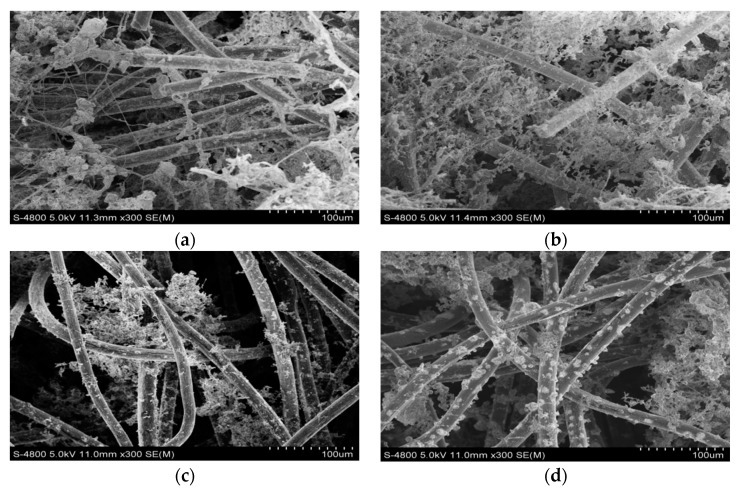
SEM images of the anode surface of three MFCs (**a**) A1; (**b**) A2; (**c**) A3 (**d**) A4.

As a corollary, the high performance of electricity generation could contribute to the large number of enriched biofilms on the carbon felt surface. In light of this, SEM analysis was conducted to make morphological observations of carbon felts supplemented with different doses of zero-valent iron. SEM images (Figure 6) recorded at high magnification clearly showed compact and dense meshes with a hierarchical structure, which had 0.5 g of zero-valent iron added (A2). Meanwhile, the biofilm of A1 was relatively porous with a looser and irregular structure and A3 or A4 showed a very loose biofilm shape, exhibiting a smaller specific surface area with no lattice-like structure. Combined with the voltage and power density discussed above, it could be concluded that carbon felt enriched with more biofilms had a better electric performance. The reason could be illustrated as follows. The parts where enriched biofilms had more exoelectrogens and electrochemically active microbes, which were comprised mainly of cytochromes, served as electron conduits between intracellular catabolic reactions and extracellular conducive materials (e.g., electrodes) [39], and had unique extracellular electron transport pathways. As we all know, some electroactive microorganisms are recognized as dissimilatory iron-reducing bacteria, which can access iron as an electron acceptor to transfer electrons during the respiration process [40]. As a result, the addition of zero-valent iron to MFC reactors could culture and catalyze the production of iron-reducing bacteria.

Zero-valent iron in the A1 reactor was the least, and the growing environment provided for microorganisms was mild and suitable, causing the acclimation of exoelectrogens and an improvement of the power generation. When the concentration of zero-valent iron was continuously increased, i.e., in the A2 reactor, microorganisms seemed to adjust the changes, so on the one hand, the enzyme activity and biological reaction rate were further improved, and on the other hand, the presence of a little amount of oxygen in the anodic reactor would gradually oxidize zero-valent iron, forming iron oxides deposited on the surface of zero-valent iron to avoid direct contact between zero-valent iron and microorganisms, which reduced the inhibitory effects on the activity of the exoelectrogens. However, organic matter would be constantly consumed by microorganisms as the dose further increased, exposing more zero-valent iron and releasing excessive iron ions, which had a toxic effect on exoelectrogens and inhibited their activity, consequently showing a rapid reduction in voltage. This phenomenon is supported by Antwi et al. [41], who pointed out that the effect of the addition of 0, 1, 4, 10, and 20 g/L ZVI on the microbial community structure was different and the microbial community shift was most pronounced in assays with 10 and 20 g/L ZVI.

### 3.5. High-Throughput Sequencing and Microbial Community Analysis

The DNA was extracted from biofilms sampled from all anodes. According to Table 2, A2 with 0.5 g of zero-valent iron added owned the highest biodiversity (Shannon index = 6.49) while A4 with no zero-valent iron added owned the lowest biodiversity (Shannon index = 5.76). So, it was indicated that supplementation of zero-valent iron could influence the distribution of the microbial community.

**Figure 7:**
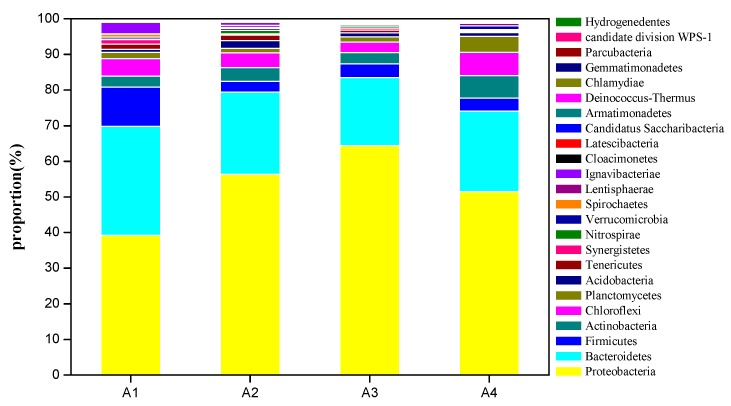
Relative abundance at the phylum level obtained with high-throughput sequencing (A1: 0.1 g zero-valent iron; A2: 0.5 g zero-valent iron; A3: 1 g zero-valent iron; A4: no zero-valent iron).

At the phylum level, bacterial species in the anode biofilm were dominated by *Proteobacteria*, *Bacteroidetes*, and *Firmicutes*, followed by *Actinobacteria*, *Chloroflexi*, *Planctomycetes*, *Acidobacteria*, and others. In the community, the *Proteobacteria* phylum was the most abundant in all samples and it was present at higher percentages in both A2 and A3 (56–64%) than other samples (39–51%). Well-known electroactive genera of this phylum (such as *Geobacter*, *Pseudomonas*, *Desulfuromonas*, etc.) often play important roles in bioelectroactive biofilms [42,43]. From Figure 7, the *Bacteroidetes* phylum was the second dominant microorganism in MFCs; it was present the most in A1 (30%) and the least in A3 (19%). The presence of *Bacteroidetes* was previously reported in fermentative bioelectrochemical biofilms due to their ability to biodegrade polymeric proteins and carbohydrates [44,45]. The *Firmicutes* phylum was often retrieved in bioelectrochemical systems, associated with the electrogenic activity. In this study, it was found in all samples: 10% for A1 and around 3% for A2, A3, and A4. Additionally, the presence of *Actinobacteria* also reflected its assistance in organic degradation since they are known to be members of versatile hydrocarbon degraders [46].

**Figure 8:**
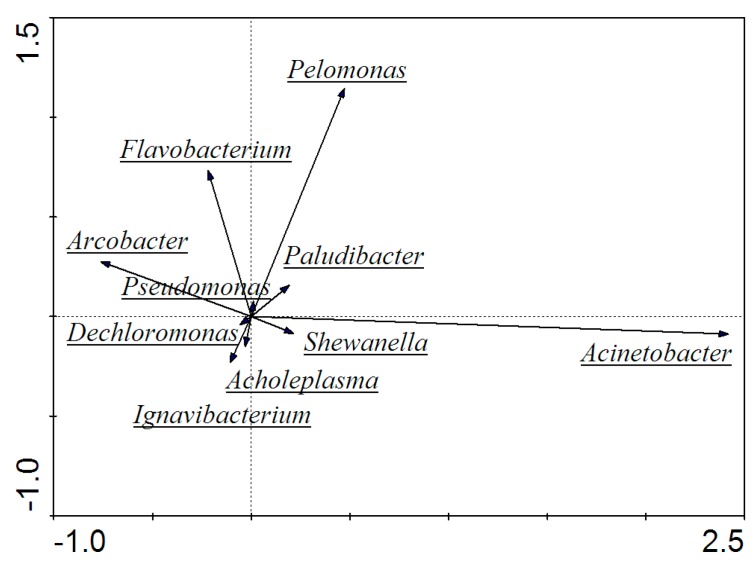
Principal component analysis (PCA) at the genus level.

Genus-level characterization further illustrated the functional roles of microbes in the MFCs community. PCA analysis of the genus level demonstrated that the dominant microorganism species were various in MFCs. Due to the wide variety of the community, microorganisms, whose relative abundance was more than 3% and possessed certain functionality, were pre-screened, and then their contributions to MFCs were judged for further analysis. From Figure 8, it was found that *Acinetobacter* was in the vicinity of first principal component axis and contributed a lot to the axis, indicating that the presence of such a microorganism directly affected the performance of the entire MFCs. In addition, *Pelomonas*, *Arcobacter*, and *Flavobacterium* were also distributed near the main coordinate axis and made such contributions to both axes, so their contents had a certain influence on MFCs to some degree. What is more, it was concluded that the gaps of the angle among the three types of microorganisms, *Pseudomonas*, *Flavobacterium*, and *Pelomonas*, were small, indicating that the three had similar properties with a certain affinity when under dosages of the zero-valent iron. Similarly, microorganisms, such as *Dechloromonas*, *Acholeplasma*, and *Ignavibacterium*, had similar gene expression ways in MFCs.

**Figure 9:**
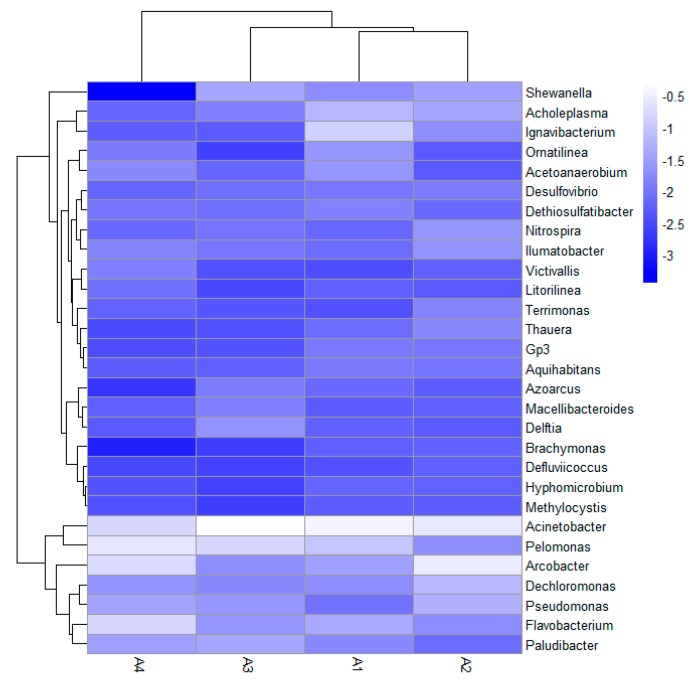
Heat map analysis of the bacterial community structures at the genus level.

Furthermore, Figure 9 shows the heat map analysis of the four reactors when different dosages of zero-valent iron were added. It could see that *Acinetobacter, Arcobacter*, and *Pelomonas* dominated in thee bacterial community while the distribution of *Shewanella* and *Ignavibacterium* in the four reactors was quite different. It is known that the heatmap package of R language can calculate the Euclidean distance between two samples by using the quantity information, and then cluster samples using the Euclidean distance. After normalizing and rearranging microorganisms, the microorganisms with similar properties will cluster while microorganisms that differ from each other will be far away. Therefore, similar conclusions could be obtained from the heat map.

Redundancy analysis (RDA) is a classification method based on the development of correspondence analysis. Combining the corresponding analysis with the multiple regression analysis, each step of the RDA is calculated with environmental factors, reflecting the relationship between microorganisms and environmental factors. Similar to the data format of PCA, five environmental factors, including voltage, OTUs, Chao, Shannon, and ACE, were selected for RDA analysis of the MFCs system. Arrows mean different environmental factors, and when the angle between environmental factors is less than 90°, this indicates there is a positive correlation between the two factors; otherwise, it is negatively correlated. What is more, the longer the line of the environmental factor, the greater the influence of the impact factor.

**Figure 10:**
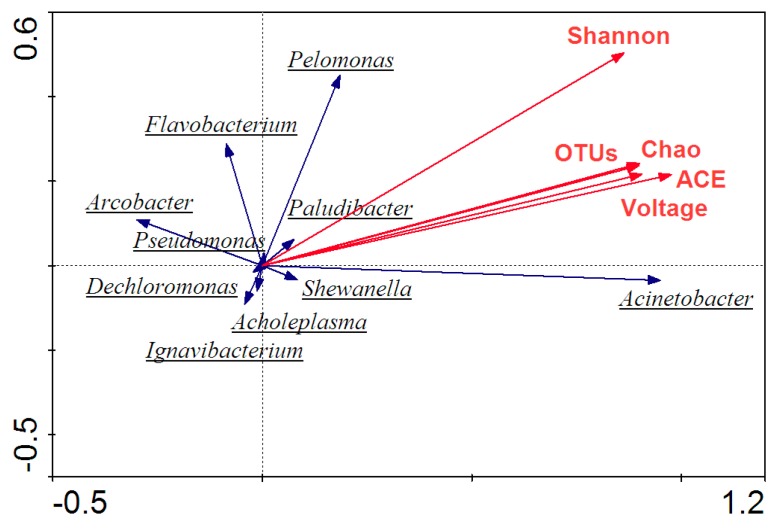
Redundancy analysis at the genus level.

In Figure 10, it was found that the angles among the voltage, OTUs, Chao, Shannon, and ACE index were less than 90°, showing a positive correlation between these environmental factors. So, it means that an increase in the abundance of microorganisms as well as community activity could synchronously enhance the electrical performance of the MFC, which is consistent with the results above. Moreover, when the relationship between the microbial community and environmental factors was further analyzed, it was revealed that gaps of the angle between the voltage and microorganisms, such as *Acinetobacter*, *Shewanella*, and *Pelomonas*, were small, showing that these species were closely related to the voltage and *Acinetobacter* had a greater contribution to the voltage.

In conclusion, the anode of the MFC is an anaerobic reactor essentially. *Acinetobacter* was reported to constitute, at least in part, the anodic bacteria in MFCs using anaerobic sludge from wastewater treatment plants as inoculate [47]. In the research of Embree et al. [48] on a single-cell genome and metatranscriptome sequencing of metabolic interactions of an alkane-degrading methanogenic community, he found that unknown members of the orders *Ignavibacteriales* became more predominant during growth on fatty acids. Therefore, there is a certain relationship between this kind of bacteria and anaerobic fermentation, which may be conducive to methane production. In addition, it was reported that the genus *Shewanella* was divided into two subgenera on the basis of the phylogenetic structure, growth properties in relation to pressure, and polyunsaturated fatty acid production [49]. Therefore, the addition of zero-valent iron changed the microbial community in the system, and further changed the anaerobic digestion process. This was mainly due to its strong reductive property, and zero-valent iron is expected to provide electrons for methanogenesis by producing water-derived H_2_ during corrosion: Fe^0^ + 2H_2_O = Fe^2+^ + 2OH^−^ + H_2_. In the above analysis, the abundance of *Acinetobacter, Ignavibacteriales*, and *Shewanella* was relatively higher compared to A4, which confirmed the practicability of zero valent iron in an anaerobic digestion system.

In terms of electricity production, *Acinetobacter* secretes an unidentified endogenous compound similar to pyrroloquinoline quinine and conducts extracellular electrontransfer in MFCs [50]. Meanwhile, it is reported that *Arcobacter* could contribute to the generation of electricity, with acetate as an electron donor, as they have been isolated from MFC reactors [51]. It was found that *Acinetobacter* distributed more in A3 and *Arcobacter* distributed more in A2, which was responsibile for the higher electrochemical performance.

Previous studies have proved the feasibility of employing *Shewanella* for bioaugmentation on MFC performance [52]. Newton et al. [53] pointed out that *Shewanella*, which is a widely applicable strain of electroactive microorganisms with a superior capacity for extracellular electron transport, was selected to represent the pure culture inoculum and to develop pure culture MFC-biosensors. Furthermore, *Shewanella* was discovered by Myers & Nealson in Lake Oneida, New York, and has been used extensively for Fe reduction in many different systems. They were the first to report the stoichiometric coupling between microbially mediated Fe reduction in clay minerals and the oxidation of organic carbon to CO_2_, using the Fe(III)-reducing bacterium *Shewanella putrefaciens* strain MR-1 with formate or lactate as the carbon source [54]. This may be the reason for the low capacity of power generation in A4 because of the lack of zero-valent iron while A2 had the best performance.

In the current research, few reports indicated the effect of zero-valent iron at the acclimation stage in MFC, most of which attempted to improve the electrical performance of MFC by modifying the electrode material or adding agent after the successful start-up of MFC. The invention, by directly adding exogenous zero-valent iron in the domestication stage, using sludge containing iron as an anode substrate to acclimate and enrich electro-producing bacteria in the anode, can not only greatly shorten the start-up time of MFC but also enhance the electricity production of the MFC. Furthermore, this study explained the basic principle of an electro-anaerobic digestion system with zero-valent iron supplementation to the MFC, which provided a practical attempt for further research. In addition, increasing the performance of power generation of the MFC by adding zero-valent iron is practically applicable, because it considers that zero-valent iron or discarded iron filings can be used as raw materials, which are low in price, highly recyclable, environmentally friendly, and in line with the essence of sustainable development.

## 4. Conclusions

Zero-valent iron was added into MFCs’ anaerobic reactor at different doses to enhance the electricity generation during the start-up stage in this work. According to the experimental results, the voltage and power density were improved by the zero-valent iron addition. When 0.5 g of zero-valent iron was added, the maximum values of the voltage and power density were 289.6 mV and 27.3 mW/m^2^, accordingly changing the structure of the biofilm and enriching the electro-active microorganisms on the anode electrode, as shown by by SEM observations. Also, carbon felt with zero-valent iron supplemented exhibited relatively superior electrochemical activity and a shifted positive peak oxidation potential as well as decreased resistance, illustrating the much better oxidizing reaction and more active metabolism of exoelectrogens on the surface of the electrode. It was observed that the quantity and diversity of microbial communities was increased, especially *Shewanella*, which are responsible for electrical generation. In the community, the *Proteobacteria* phylum was the most abundant in all samples and it was present at higher percentages in both A2 and A3 (56–64%) than other samples (39–51%). These results suggested that the performance and anerobic biological community of MFCs can be improved by adding zero-valent iron.

## Figures and Tables

**Table 1:** The Alpha diversity of the four activated sludge reactors.

	R_S_/Ω	R_CT_/Ω × 10^−3^	C/F × 10^−3^	W/(S•s^−0.5^) × 10^−3^
A1	18.44	25.600	0.0290	1.53
A2	24.71	10.000	27.9	11.9
A3	22.76	32.470	2.26	0.330
A4	23.98	33,840	1.12	23.8
primary	22.24	35,730	0.360	70.2

**Table 2:** Analysis of diversities according to high-throughput sequencing.

	OTUs	Chao	Shannon	ACE	Simpson
A1	3495	10,386.96	6.104079	17,856.5333	0.011626
A2	4578	15,520.60	6.492959	29,822.3437	0.013034
A3	3539	10,659.00	5.786235	18,800.1165	0.026910
A4	2762	6914.500	5.765082	10,409.8210	0.020122
